# A case report of gastric antral vascular ectasia treated by endoscopic band ligation combined with lauromacrogol injection

**DOI:** 10.1097/MD.0000000000041235

**Published:** 2025-01-24

**Authors:** Linbo Chen, Keke Sun, Yukai Chen, Pingping Hu, Qi Lin

**Affiliations:** a Department of Gastroenterology, The Affiliated People’s Hospital of Ningbo University, Ningbo, China.

**Keywords:** case report, endoscopic band ligation, esophagogastroduodenoscopy, gastric antral vascular ectasia, lauromacrogol injection

## Abstract

**Rationale:**

Gastric antral vascular ectasia (GAVE) is a rare acquired lesion characterized by vascular dilation in the gastric antrum, frequently results in occult or overt gastrointestinal bleeding. Endoscopic intervention remains the cornerstone of therapy. Argon plasma coagulation was previously considered a first treatment option. But recently, endoscopic band ligation (EBL) has emerged as an alternative, increasingly favored for its safety and efficacy. Nonetheless, a consensus on the most effective treatment approach has yet to be established.

**Patient concerns:**

A 74-year-old female was hospitalized for persistent chest tightness and dyspnea for 1 year. Physical examination showed an anemic appearance with normal blood pressure. Upon admission to the hospital, the blood routine examination revealed severe anemia and the fecal occult blood test was persistently positive.

**Diagnoses:**

The endoscopic observations and histological evidence led to a diagnosis of GAVE for the patient.

**Interventions:**

Considering the poor response to prior pharmacotherapy, endoscopic intervention was selected for this hospitalization. The initial EBL alone did not yield particularly satisfactory results. Combining EBL with lauromacrogol injection as a subsequent treatment resulted in encouraging outcomes.

**Outcomes:**

At the 6-week follow-up, the patient exhibited a negative fecal occult blood test, normalization of hemoglobin level, and endoscopic images demonstrated near complete resolution of vascular ectasias.

**Lessons:**

The combination of EBL with lauromacrogol injection has shown a satisfactory short-term outcome, providing a new option for the endoscopic management of GAVE. However, its long-term efficacy still requires further observation.

## 1. Introduction

Gastric antral vascular ectasia (GAVE), a rare etiology of upper gastrointestinal bleeding, contributes to approximately 4% of non-variceal upper gastrointestinal hemorrhages.^[1]^ Endoscopic intervention is the first-line treatment option for GAVE, with argon plasma coagulation (APC) previously being the predominant modality.^[2]^ Recent studies have demonstrated the superiority of endoscopic band ligation (EBL) on APC for GAVE management.^[3,4]^ However, literature on combined endoscopic approaches for GAVE remains scarce. In this article, we describe a case of a patient with GAVE-induced recurrent anemia who initially failed to achieve optimal results with EBL monotherapy. Subsequent treatment combining EBL with lauromacrogol injection led to a satisfactory outcome. The patient currently exhibits no signs of active gastrointestinal bleeding, maintains normal hemoglobin levels, and a follow-up esophagogastroduodenoscopy (EGD) reveals substantial reduction in the antral lesions.

## 2. Case presentation

### 2.1. Chief complaints

A 74-year-old female patient presented with persistent chest tightness and dyspnea persisting for 1 year.

### 2.2. History of present illness

The patient had persistent chest tightness and dyspnea for 1 year without special treatment. She reported no abdominal pain, hematemesis, melena, or hematochezia.

### 2.3. History of past illness

She had the history of well-controlled hypertension and hyperlipidemia. She denied any hepatic diseases or autoimmune disorders.

### 2.4. Personal and family history

The patient never smoked or drank. There was no family history of malignant tumors.

### 2.5. Physical examination

Physical examination showed anemic appearance and sinus tachycardia with normal blood pressure. Abdominal palpation detected no masses. No palpable enlargement of the liver and spleen. Rectal examination did not reveal blood in stools. Body mass index was 30.4 kg/m^2^.

### 2.6. Laboratory examinations

Laboratory data evidenced that the patient presented with microcytic hypochromic anemia, as indicated by a hemoglobin level of 5.7 g/dL (reference range 11.5–15.5 g/dL). Following a blood transfusion, her hemoglobin increased to 8.2 g/dL. Iron studies demonstrated a ferritin level of 7.54 ng/mL (reference range 13–318 ng/mL), serum iron of 1.9 μmol/L (reference range 7.8–32.2 μmol/L) and a transferrin saturation of 1.7% (reference range 20%–55%). The reticulocyte count was elevated at 2.5% (reference range 0.5%–1.5%). Bone marrow examination disclosed no aberrant cell populations, aligning with the diagnosis of microcytic hypochromic anemia. Further laboratory evaluations showed an antinuclear antibody titer of 1:1000 and an elevated gastrin level of 769 ng/L (reference range 13–115 ng/L). The patient had multiple positive fecal occult blood tests (FOBT). Other significant laboratory test results, including alanine aminotransferase, aspartate aminotransferase, platelet counts, and coagulation function, fall within the normal range.

### 2.7. Imaging examinations

Abdominal computed tomography scan showed that the patient had a fatty liver. Cardiac ultrasound and chest computed tomography scan were normal.

### 2.8. Further diagnostic work-up

**Figure 1.**
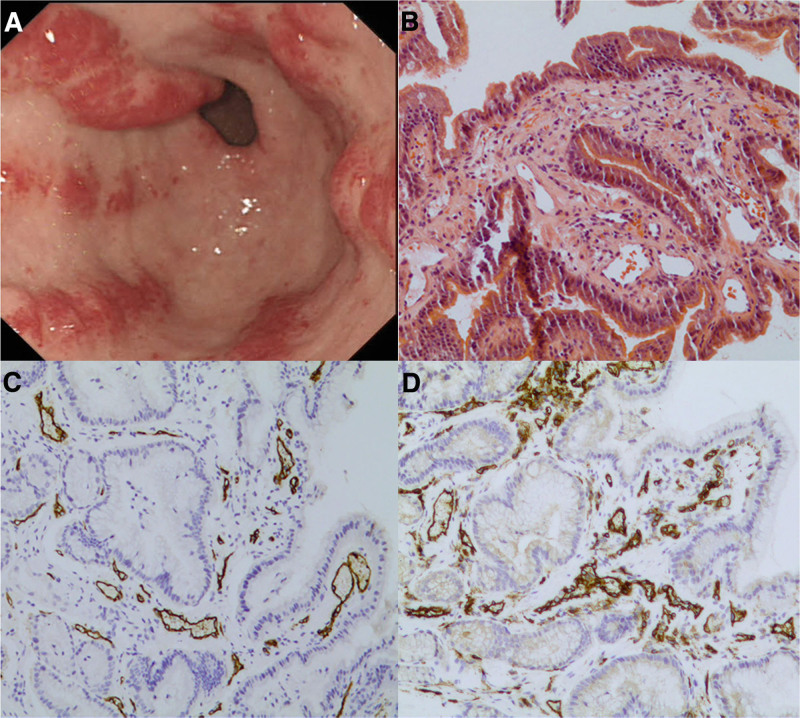
Endoscopic findings and histological biopsy of the patient on admission to hospital; radial erythema was observed in the gastric antrum (A), hematoxylin and eosin (H&E) staining revealed dilated capillaries (B), and immunohistochemistry showed positive staining for CD31 (C) and CD34 (D).

During a subsequent EGD accompanied by histological biopsy, radial erythema was observed in the gastric antrum, with some areas showing nodular elevations (Fig. 1A). Hematoxylin and eosin staining revealed the presence of dilated capillaries (Fig. 1B), whereas immunohistochemical analysis indicated positive staining for CD31 (Fig. 1C) and CD34 (Fig. 1D). Colonoscopy examination did not show any obvious lesions.

### 2.9. Final diagnosis

The endoscopic observations and histological evidence led to a diagnosis of GAVE for the patient.

### 2.10. Treatment

**Figure 2.**
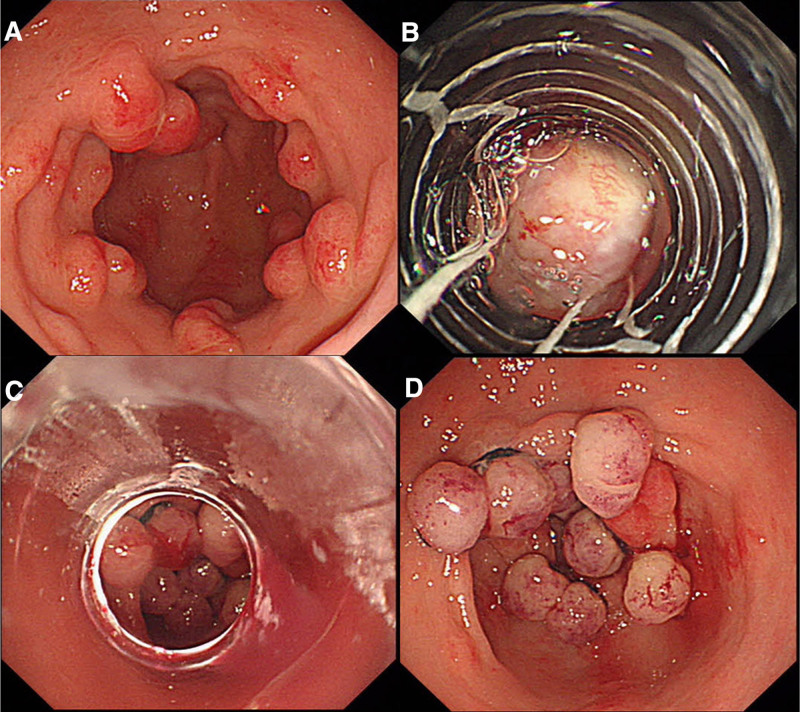
Treatment of gastric antral vascular ectasia with endoscopic band ligation in the first operation (A–D).

**Figure 3.**
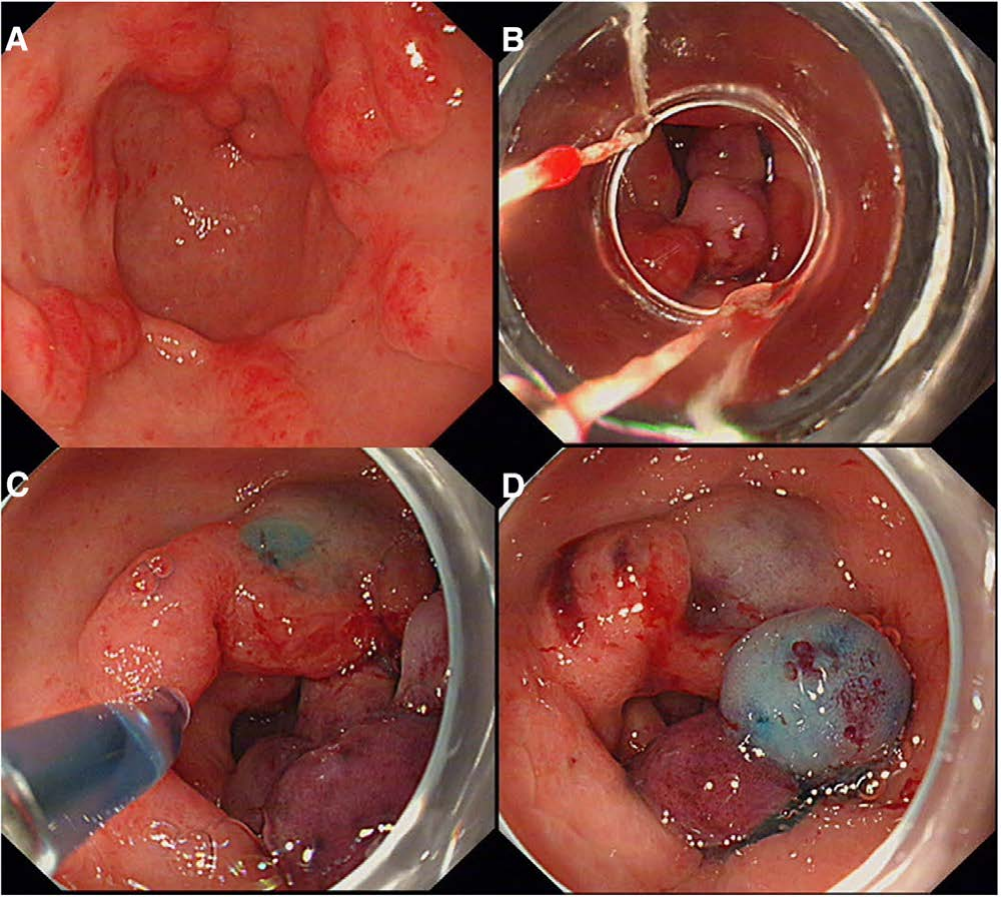
Treatment of gastric antral vascular ectasia with endoscopic band ligation plus lauromacrogol injection in the second operation (A–D).

Considering the poor response to prior pharmacotherapy, we selected endoscopic intervention for this patient. Given the patient’s high expectations for treatment and the preference to reduce the risk of recurrence, we opted for EBL based on recent research findings. We utilized a multi-band ligator equipped with 10 bands to treat the radial erythematous stripes emanating from the distal gastric antrum, covering the majority of the lesion (Fig. 2). The procedure was uneventful with no intraoperative or postoperative complications, and the patient did not complain of any significant discomfort. Discharge occurred 48 hours post-procedure, with ongoing administration of omeprazole and rebamipide. Hematology assessment at 6 weeks after discharge revealed an increase in hemoglobin levels to 11.3 g/dL, but the FOBT still positive. Subsequent endoscopy indicated a reduction in lesions within the gastric antrum compared to the initial presentation 6 weeks earlier. However, persistent mucosal erythema and nodularity suggested a suboptimal therapeutic response. Consequently, we innovatively performed a combination of EBL with lauromacrogol injection as a treatment strategy for the patient. During the operation, we reapplied EBL to the areas with radial stripes and administered lauromacrogol injections to the more severe parts of the lesions (Fig. 3). This second intervention mirrored the first in its lack of complications and the absence of discomfort for the patient, who was again discharged 48 hours post-procedure.

### 2.11. Outcome and follow-up

**Figure 4.**
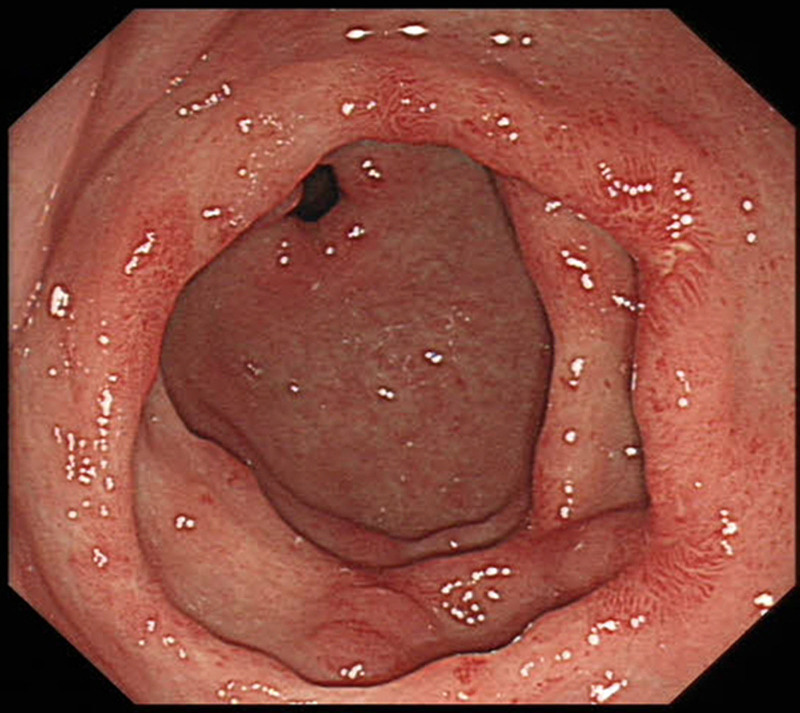
Follow-up esophagogastroduodenoscopy 6 weeks after the second operation showed reduction of the lesion and scar formation.

The patient currently has no symptoms of chest tightness or dyspnea. At the 6-week follow-up, the patient exhibited a negative FOBT, normalization of hemoglobin levels to 12.9 g/dL, and endoscopic images demonstrated near complete resolution of vascular ectasias (Fig. 4).

## 3. Discussion

GAVE, a rare disease characterized by dilated blood vessels in the antrum radiating to the pylorus, was first described by Rider et al in 1953.^[5]^ Advancements in endoscopic techniques have led to further characterization of GAVE in 1984, with Jabbari et al^[6]^ naming it “watermelon stomach” due to its characteristic pattern similar to the stripes observed on watermelons. The pathophysiology of GAVE remains elusive and it was thought to be associated with chronic liver disease and autoimmune and connective tissue diseases.^[7]^ The patient exhibited elevated total antinuclear antibodies levels, yet showed no evidence of diagnosable autoimmune and connective tissue diseases. Recent studies increasingly suggest a correlation between GAVE and metabolic syndrome,^[8]^ including obesity, hyperlipidemia, hypertension, diabetes, and nonalcoholic fatty liver disease.^[9,10]^ The patient’s obesity, hypertension, and hyperlipidemia lend further support to this association. Additionally, the patient presented with hypergastrinemia, but the role of gastrin in the etiology of GAVE remains controversial.^[11,12]^

**Figure 5.**
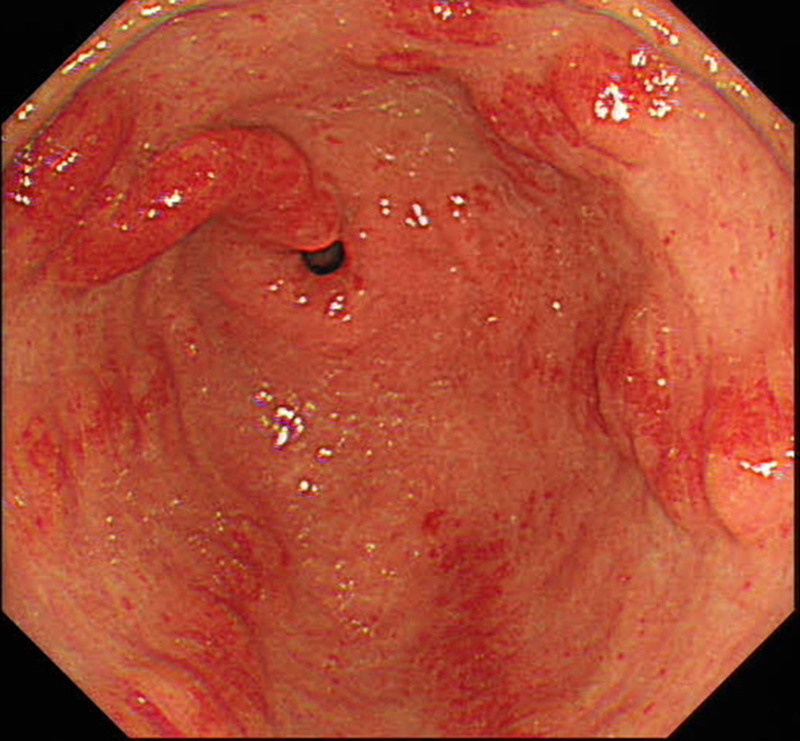
Esophagogastroduodenoscopy 1 year ago.

**Figure 6.**
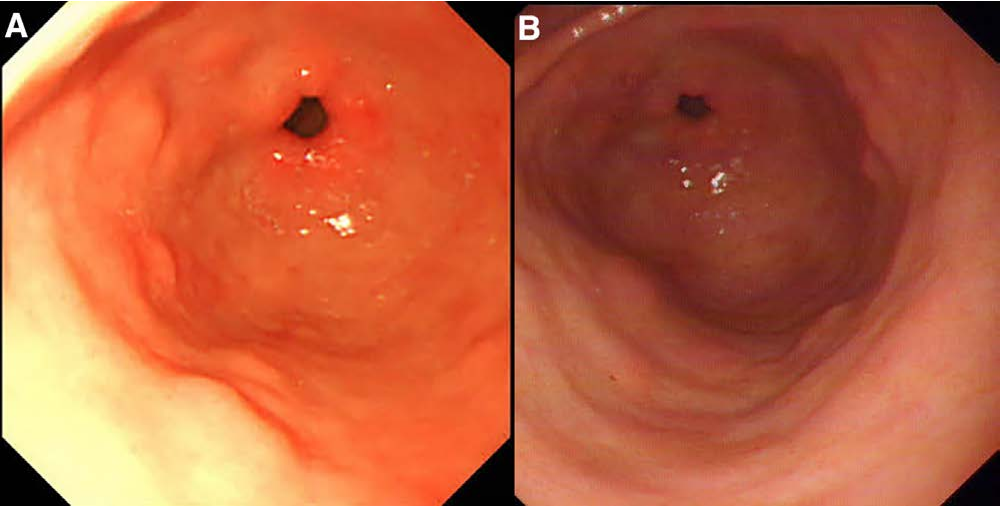
The esophagogastroduodenoscopy 7 years ago (A) and 6 years ago (B) did not show any typical endoscopic findings of gastric antral vascular ectasia.

GAVE can manifest in multiple patterns based on endoscopic findings, with the 3 common subtypes being “watermelon stomach,” “honeycomb stomach,” and the more recently described nodular GAVE,^[13]^ which in this case can be classified as “watermelon stomach.” Notably, approximately 40% of GAVE cases are endoscopically misdiagnosed, often confused with erythema, polyps, gastritis, and ulcers.^[13]^ Among the 3 GAVE categories, nodular GAVE is most susceptible to misclassification. Due to its endoscopic resemblance to hyperplastic polyps and the challenges in histological differentiation, nodular GAVE frequently receives incorrect diagnoses.^[14]^ While histopathological analysis can assist in accurate diagnosis, it is compromised by a high false-negative rate due to inadequate sample sizes, and thus, should not serve as the sole diagnostic criterion.^[15,16]^ The important value of endoscopic evaluation should not be overlooked. A year prior, the patient exhibited characteristic endoscopic signs of GAVE (Fig. 5), yet the condition was misidentified as chronic erosive gastritis. Despite treated with medication, the patient experienced persistent anemia and positive FOBT over the subsequent year. Therefore, enhancing the diagnostic ability for GAVE and selecting efficacious therapeutic strategy are essential. The EGDs conducted 7 years ago (Fig. 6A) and 6 years ago (Fig. 6B) revealed no classic endoscopic indications of GAVE, only mild erythema was observed in the gastric antrum. This suggests that the patient’s gastric antral lesions have evolved over time, aligning with the concept that GAVE is an acquired disease.^[11]^ Regrettably, no histological biopsy was performed at that time.

At present, no pharmacological agent has been conclusively demonstrated as effective for treating GAVE.^[17]^ Endoscopic intervention is still the cornerstone of management. The decision to treat and the choice of therapeutic approaches primarily hinges on the preferences of endoscopist. Previous endoscopic treatment modalities including heater probe, bipolar electrocoagulation, Nd-YAG laser and cryotherapy have been largely replaced by APC due to lower success rates and issues of availability.^[2,18]^ Nevertheless, large variability in endoscopic success rates and high recurrence rates limit the effectiveness of APC in clinical practice. EBL has recently emerged as an alternative, increasingly favored for its safety and efficacy.^[4]^ Studies have shown that patients treated with EBL experience a more significant endoscopic response than those treated with APC, benefiting from reduced posttreatment transfusion requirements and fewer follow-up interventions.^[19–21]^ Based on the histological characteristics of GAVE, which include dilated and tortuous submucosal veins accompanied by extensive mucosal lesions, it can be inferred that targeting deeper layers may yield improved outcomes. APC is a superficial technique act mostly on the mucosal layer, while EBL is a deep submucosal technique can act on the mucosal layer and submucosal layer.^[17]^ Neil et al suggest that EBL may be preferentially selected for more severe cases of GAVE,^[4]^ possibly because EBL affects deeper layers of the gastric wall, leading to thrombosis and ischemia of the mucosa and submucosa, thus providing a more comprehensive hemostatic effect.^[22]^

EBL is a well-established technique in clinical practice, with its safety and feasibility widely confirmed across various conditions, including esophageal varices, Dieulafoy lesions, angiodysplasia, blue rubber vesicle nevus syndrome and hemorrhoids.^[23–26]^ In the present case, EBL was utilized for GAVE during the first operation, employing a Multi-Band Ligator equipped with 10 bands. A follow-up EGD 6 weeks post-procedure showed that the lesion reduction was not particularly noticeable. Although there was an increase in the patient’s hemoglobin levels, but the FOBT remained positive. Therefore, we wanted to explore a more effective treatment modality. Combined endoscopic therapy have demonstrated advantages in managing esophageal varices,^[27–29]^ but reports on GAVE treatments are scarce. To date, only Chen et al^[30]^ reported that APC in conjunction with sclerotherapy can lower recurrence rate, while EBL plus sclerotherapy injection for treating GAVE has not yet been reported. After obtaining informed consent, we performed EBL for most of the gastric sinus lesions during the second operation, while lauromacrogol injections were administered to the relatively more severe lesions. Currently, blood routine reexamination at 6 weeks after procedure showed hemoglobin has risen to normal level, the FOBT was negative, and a follow-up EGD indicated considerable lesion shrinkage, denoting a satisfactory short-term outcome. However, its long-term efficacy still warrants further observation. The patient has been advised to undergo subsequent EGD, blood routine analysis, and FOBT after 6 months and 1 year to evaluate the long-term outcome.

## 4. Conclusion

GAVE is a rare clinical disease which can cause severe upper gastrointestinal tract bleeding. Nonetheless, a consensus on the most effective treatment approach has yet to be established. The combination of EBL with lauromacrogol injection has shown a satisfactory short-term outcome, providing a new option for the endoscopic management of GAVE. However, its long-term efficacy still requires further observation.

## Acknowledgements

We sincerely appreciate the patient for her cooperation in information acquisition, treatment, and follow-up.

## Author contributions

**Conceptualization:** Qi Lin.

**Investigation:** Yukai Chen.

**Methodology:** Keke Sun.

**Project administration:** Qi Lin.

**Writing – original draft:** Linbo Chen.

**Writing – review & editing:** Pingping Hu.
